# Low-Computational Cost Stitching Method in a Three-Eyed Endoscope

**DOI:** 10.1155/2019/5613931

**Published:** 2019-06-17

**Authors:** Virginia Mamone, Sara Condino, Fabrizio Cutolo, Izadyar Tamadon, Arianna Menciassi, Michele Murzi, Mauro Ferrari, Vincenzo Ferrari

**Affiliations:** ^1^Information Engineering Department, University of Pisa, Pisa 56122, Italy; ^2^EndoCAS Center for Computer-Assisted Surgery, Pisa 56124, Italy; ^3^Sant'Anna School of Advanced Studies, Pisa 56127, Italy; ^4^Department of Adult Cardiac Surgery, G. Pasquinucci Heart Hospital, Gabriele Monasterio Foundation, Massa 54100, Italy; ^5^Department of Translational Research and New Technologies in Medicine and Surgery, University of Pisa, Pisa, Italy

## Abstract

Aortic valve replacement is the only definitive treatment for aortic stenosis, a highly prevalent condition in elderly population. Minimally invasive surgery brought numerous benefits to this intervention, and robotics recently provided additional improvements in terms of telemanipulation, motion scaling, and smaller incisions. Difficulties in obtaining a clear and wide field of vision is a major challenge in minimally invasive aortic valve surgery: surgeon orientates with difficulty because of lack of direct view and limited spaces. This work focuses on the development of a computer vision methodology, for a three-eyed endoscopic vision system, to ease minimally invasive instrument guidance during aortic valve surgery. Specifically, it presents an efficient image stitching method to improve spatial awareness and overcome the orientation problems which arise when cameras are decentralized with respect to the main axis of the aorta and are nonparallel oriented. The proposed approach was tested for the navigation of an innovative robotic system for minimally invasive valve surgery. Based on the specific geometry of the setup and the intrinsic parameters of the three cameras, we estimate the proper plane-induced homographic transformation that merges the views of the operatory site plane into a single stitched image. To evaluate the deviation from the image correct alignment, we performed quantitative tests by stitching a chessboard pattern. The tests showed a minimum error with respect to the image size of 0.46 ± 0.15% measured at the homography distance of 40 mm and a maximum error of 6.09 ± 0.23% at the maximum offset of 10 mm. Three experienced surgeons in aortic valve replacement by mini-sternotomy and mini-thoracotomy performed experimental tests based on the comparison of navigation and orientation capabilities in a silicone aorta with and without stitched image. The tests showed that the stitched image allows for good orientation and navigation within the aorta, and furthermore, it provides more safety while releasing the valve than driving from the three separate views. The average processing time for the stitching of three views into one image is 12.6 ms, proving that the method is not computationally expensive, thus leaving space for further real-time processing.

## 1. Introduction

In recent years, the main risk factors for heart disease, such as smoking, high cholesterol, and high blood pressure, have increased. Aortic stenosis (AS) is the third most prevalent form of cardiovascular disease in the Western world, with a 50% risk of death in the three years following the onset of symptoms [1]. AS develops from progressive calcification of leaflets, reducing the leaflet opening over time, until the valve is no longer able to control the blood flow. It is a highly prevalent condition, being present in 21–26% of elderly above 65 [2] and no pharmacologic treatment showed to be effective, nor attenuating the progressive valve calcification, nor improving survival [3]. The only definitive treatment for AS in adults is aortic valve replacement (AVR). For decades, surgical AVR has been the standard treatment for severe AS. The traditional open-heart method gives the surgeon direct access to the heart through median sternotomy. But despite allowing excellent access to all cardiac structures, the open-heart method requires complete division of the sternum and sternal spreading, thus disrupting the integrity of the chest wall in the early recovery phase. Since the first surgical AVR intervention in 1960, less invasive methods have been investigated to complete the operation. In 1997 and 1998, surgeons performed the first intervention, respectively, in right mini-thoracotomy and mini-sternotomy [4, 5]. A few years later, a percutaneous transcatheter approach was tested, which to date is the standard intervention for high-risk patients. However, for low-risk patients, this approach is not advisable due to the increase in the chance of paravalvular regurgitation, implantation of pacemaker, and a worst 3-year survival [6].

For many patients, the best solution remains surgery via mini-thoracotomy or mini-sternotomy. These techniques offer the typical benefits of minimally invasive surgery, such as decrease in blood transfusion, hospital stay, and improved cosmesis, demonstrated not only in the cardiothoracic district but also in the vascular one [7]. Through robotics, further benefits can be reached in terms of telemanipulation, motion scaling, and even smaller incisions [8, 9]. Researchers proposed various robotic systems to assist heart surgery [10], allowing in some cases the preoperative planning to test the surgical case before the actual intervention [11].

A major challenge of minimally invasive techniques, including those based on a robotic solution, remain visualization: the surgeon lacks the direct view of the operative field and has a poor spatial awareness in the rather limited available space. Augmented reality is a promising asset in the context of image-guided surgery [12–14]. In endoscopic techniques, surgeon usually focuses on a stand-up monitor to perform the surgical task. Indeed, in such procedures, the surgeon operates watching endoscopic video images reproduced on the spatial display unit. Therefore, in endoscopic surgery, the augmented information is obtained by merging endoscopic video frames with virtual content useful for increasing spatial awareness and for aiding the surgical tasks [15]. Recent in vitro and cadaver studies have proven the efficacy of augmented reality in assisting endoscopic surgery [16–20].

To increase the field of view and offer a wider vision of the operative field, some solutions suggest the use of two cameras and the application of stitching techniques to merge the pairs of images into one [21]. However, traditional methods for stitching images such as the SIFT [22], SURF [23], or ORB [24] algorithms are computationally expensive, because they require the identification of features in each image and the search for correspondences between each image pair. This makes these powerful and effective methods restraining in real-time application. Other solutions consist of expanding the surgeon's field of view through dynamic view expansion: in a recent work, images from a single camera are merged using simultaneous localization and mapping (SLAM) to generate a sparse probabilistic 3D map of the surgical site [25]. The problem of visualization in minimally invasive systems is substantial, and several companies already provide systems such as the Third Eye Retroscope and Third Eye Panoramic, which allow framing larger areas through auxiliary systems, or the Fuse (full-spectrum endoscopy), a complete colonoscopy platform including a video colonoscope and a processor. The systems currently on the market, however, are mostly developed for colonoscopy or gastroscopy and cannot be integrated for use in heart operations due to the different morphology and surgical task.

This work focuses on the development of a computer vision methodology to increase the field of view and offer a wider vision of the operative field. The proposed methodology can be used for the navigation of any minimally invasive instrumentations, including robotic systems.

In this paper, the proposed approach was tested for the guidance of the robotic system for minimally invasive valve surgery developed in [26]. The robot is a flexible manipulator, having omnidirectional bending capabilities. Endoscopic vision through three small cameras on the robot tip is aimed to aid the surgeons in accurately positioning the aortic valve. The objective of this work was to evaluate the best way to merge the information coming from the cameras in real time, making it easier for the surgeon to orientate through the operative field. At the same time, it was also necessary to contain the computational cost to allow real-time operation while including the required computations to other device features.

## 2. Materials and Methods

The following sections describe computer vision issues to implement an image stitching method for a generic three-eyed endoscopic system. Then, the specific image-guided robotic platform for minimally invasive aortic valve replacement is presented together with the setup used to perform preliminary test.

### 2.1. Image Stitching for Three-Eyed Cameras Endoscope

The proposed approach was developed for three-eyed endoscopic systems, which compared to classical 2D monoview endoscopic instruments offer improved navigation functionalities since it can allow for triangulation and stereo reconstruction and can offer a wider vision of the operative field. In these systems, however, the different off-axis viewpoints provide visual information not as usable if compared to the usual endoscopic view, where a single camera is centered on the workspace. This can be aggravated by the fact that, to facilitate the other functionalities, the cameras can be nonparallel oriented.

The proposed image stitching method can be performed by applying an appropriate image warping based on the estimation of the three plane-induced homographies between each camera and a virtual camera placed at their barycenter and oriented as one of them, chosen as a reference.

**Figure 1:**
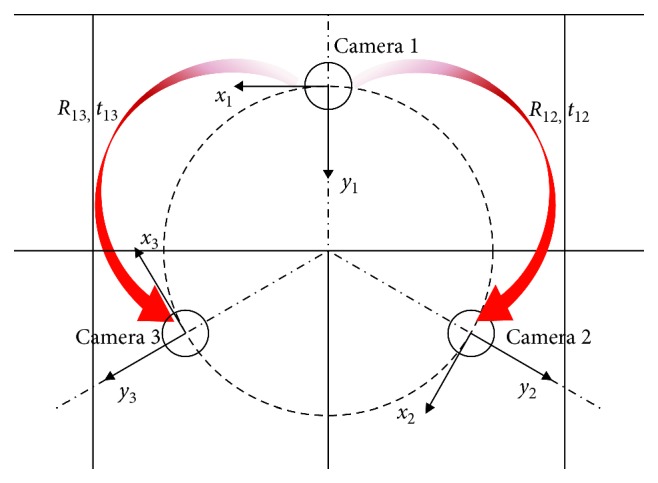
Possible camera configuration in an endoscopic instrument. The reference systems of the three cameras are oriented radially with respect to the manipulator axis. Camera 1 is the reference camera.

Figure 1 shows a possible camera configuration in an endoscopic instrument with a central operative lumen. We will refer to the three cameras with numbers from 1 to 3, number 1 being associated with the reference camera.

The following paragraphs describe the steps employed to achieve an accurate and reliable image stitching: we start from the description of the employed methods for camera calibration, and then, we introduce the basic concept of homographic transformations before describing the employed image stitching procedure.

#### 2.1.1. Camera Calibration

Camera calibration, which involves the estimation of the camera intrinsic and extrinsic parameters, is the first essential procedure.

Plane-based camera calibration methods, as the well-known Zhang's method [27], which requires the camera to observe a planar calibration pattern at a few unknown orientations can be applied. At first, the matches between 3D world points (corners of a given chessboard) and their corresponding 2D image points are found. For this purpose, in this work, we used a 4 × 5 chessboard calibration pattern with 5 mm square side.

To estimate the matrix of the intrinsic linear parameters, *K*_*i*_, and the radial distortion coefficients, the intrinsic calibration is carried out for each camera, *i*. In our method, we proposed the use of a radial distortion model with only two coefficients, neglecting the tangential distortion. The MATLAB calibration toolbox, which allows the selection of the most appropriate images and the elimination of any outliers based on the retroprojection error, was used. We ensured that all image areas were covered by the grid, to get the most accurate estimate of the distortion parameters.

After the estimation of the three cameras' intrinsic parameters, the extrinsic calibration is performed to get the relative poses of the three cameras: this phase allows estimating the reciprocal poses of the cameras, minimizing the total reprojection error for all the corner points in all the available views from the camera pairs. This phase can be performed in C++ language using the OpenCV libraries. The substantial advantage of using this environment is the possibility to perform the calibration whilst keeping the intrinsic parameters obtained in the previous calibration as fixed. By doing so, the estimation of the extrinsic parameters can be more accurate than estimating intrinsic parameters and relative poses together; the high dimensionality of the parameter space and the low signal-to-noise ratio in the input data can cause the function to diverge from the correct solution. Also, working with three pairs of cameras and dealing with the estimation of three reciprocal poses, it is essential to use univocal intrinsic parameters, so that the resulting poses are consistent.

The output of this step is two rigid transformations, in form of rototranslation matrices, relating the reference systems of camera 2 and camera 3 with respect to camera 1 reference system. As highlighted in red in Figure 1, in the following paragraph, we will refer to the following:

- *R*_12_ and *t*_12_ as the rotation and translation component of the rigid transformation from camera 1 to camera 2
- *R*_13_ and *t*_13_ as the rotation and translation component of the rigid transformation from camera 1 to camera 3

#### 2.1.2. Homographic Transform

A plane-induced homography is a projective transformation that relates the images of a reference plane in the world, grabbed by two generic cameras placed at different positions and/or orientations. Such homography describes the pixel-to-pixel relation between two camera images, *x*_*i*_ and *x*_*j*_, as follows:

(1) $$\begin{array}{c} \lambda x_{j}=H_{ij}\left(R_{ij},\;t_{ij},\;K_{i},\;K_{j},\;\pi \right)x_{i}. \end{array}$$

The image points from two cameras, *x*_*i*_ and *x*_*j*_, are expressed in homogeneous coordinates, and *λ* is the generic scale factor due to the equivalence of homogeneous coordinate rule. The homography is a function of the relative pose between the two cameras (*R*_*ij*_, *t*_*ij*_), the intrinsic parameters of the two cameras (*K*_*i*_, *K*_*j*_), and the position and orientation of the reference plane in the scene with respect to the camera *i*. *H*_*ij*_ can be broken down as follows:

(2) $$\begin{array}{c} H_{ij}=K_{j}\left(R_{ij}+\frac{t_{ij}\cdot n_{i}^{\prime }}{d_{i}}\right)K_{i}^{-1}, \end{array}$$

where *n* is the normal unit vector of the reference homography plane with respect to the camera *i* and *d* is the distance between the origin of the camera *i* reference system and the plane.

#### 2.1.3. Image Stitching

Image stitching can be performed by applying an appropriate warping of the camera images based on the estimation of the three plane-induced homographies between each camera image and a virtual camera, ideally placed at their barycenter [28]. This allows us to remap each camera view on an ideal and central viewpoint of the operatory site plane.

The three homographic transformations that relate the views of each camera to the virtual camera are

(3) $$\begin{array}{c} H_{1\text{v}}=K_{\text{v}}\left(R_{1\text{v}}+\frac{t_{1\text{v}}\cdot n_{1}^{\prime }}{d_{1}}\right)K_{1}^{-1},\\ H_{2\text{v}}=K_{\text{v}}\left(R_{2\text{v}}+\frac{t_{2\text{v}}\cdot n_{2}^{\prime }}{d_{2}}\right)K_{2}^{-1},\\ H_{3\text{v}}=K_{\text{v}}\left(R_{3\text{v}}+\frac{t_{3\text{v}}\cdot n_{3}^{\prime }}{d_{3}}\right)K_{3}^{-1}, \end{array}$$

where *K*_v_ represents the intrinsic matrix of the virtual camera, which is associated with a wider field of view than the single cameras to encompass all three individual views. The parameters *n*_*i*_ and *d*_*i*_ differ, although they refer to the same plane, as both are relative to the associated real camera reference system.

The rototranslations relating the poses of each camera to the virtual camera are provided by the calibration as follows:

(4) $$\begin{array}{c} R_{1\text{v}}=I,\\ t_{1\text{v}}=-b,\\ R_{2\text{v}}=R_{12}^{-1},\\ t_{2\text{v}}=-R_{12}^{-1}t_{12}-b,\\ R_{3\text{v}}=R_{13}^{-1},\\ t_{3\text{v}}=-R_{13}^{-1}t_{13}-b, \end{array}$$

where *b* is the center of gravity position in the camera 1 reference system.

### 2.2. Three-Eyed Cameras Endoscopic Vision for Robotic Aortic Valve Replacement

In minimally invasive heart valve surgery, surgeons can replace the aortic valve through a small incision (less than 40 mm) between the ribs. Controllable flexible manipulators, with appropriate endoscopic vision system, are ideal for accessing such areas of the patient's chest through a small entry point.

This application requires a great deal of accuracy in reaching and targeting the proper site of valve implantation, the annulus plane (the cross section with smallest diameter in the blood path between the left ventricle and the aorta). This system can greatly take advantage from the use of multicameras imaging system, with triangulation functionalities to extract in real-time the 3D position of the anatomical target and image stitching functionalities to offer the surgeon with a wider vision of the operative field.

**Figure 2:**
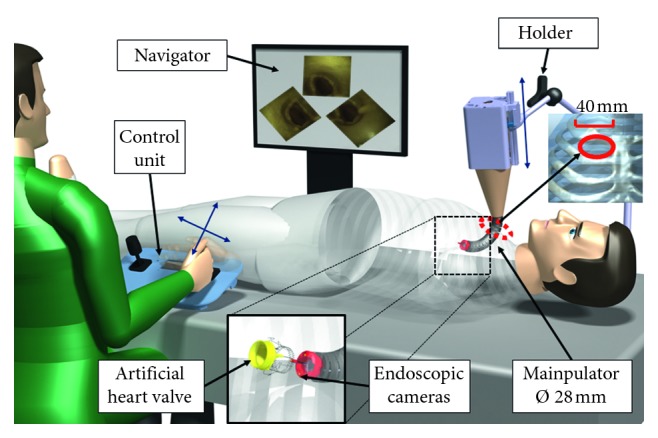
Proposed platform for aortic heart valve surgery. The camera images are processed and presented to the surgeon for navigation. The surgeon uses the joysticks in the control unit to operate the robot, which is held in place by the holder.

The robotic platform is described in [26].  Figure 2 shows an overall view of the image-guided robotic system: the surgeon controls the robot through joysticks integrated in the control unit, and the navigation is guided by three camera views.

The robotic system is a 5-DoF cable-driven flexible manipulator with internal introducer and a visualization aid, named navigator in Figure 2.

In the proposed surgical scenario, the flexible manipulator is attached to a linear actuator and is held fixed by a holder, attached to the patient's bed. The flexible manipulator has omnidirectional bending capabilities which are controlled by 4 set of cables and servomotors. The flexible part is 130–150 mm long with an external diameter of 28 mm and up to 120 degrees of maximum bending.

**Figure 3:**
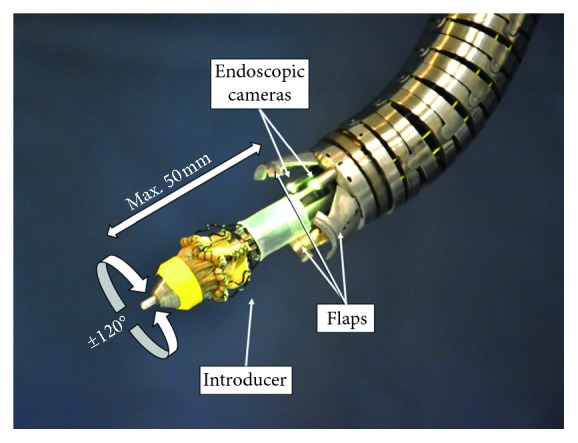
The manipulator. The robotic manipulator exposes the valve preloaded on the introducer. The maximum valve release distance is 50 mm, while the maximum rotation angle is 120 degrees.

**Figure 4:**
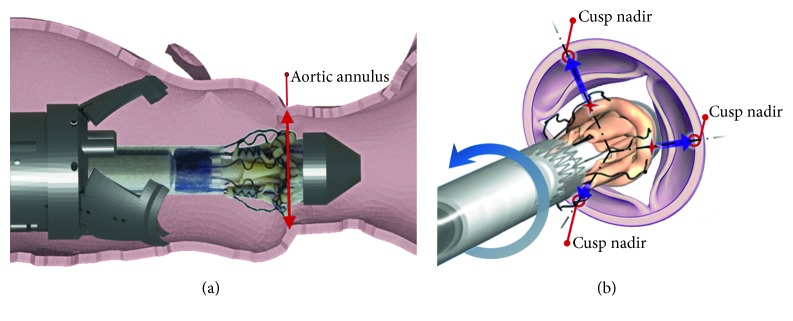
Correct positioning of the introducer. (a) The introducer is aligned with the plane corresponding to the aortic annulus, where the nadirs of the cusps reside. (b) Following a rotation on its own axis, the introducer is oriented to match the nadir of the replacement valve with the nadir of the old calcified valve.

The surgical procedure requires a cardiopulmonary bypass that provides a bloodless field for the intervention. Next the manipulator, shown in Figure 3, is inserted into the aorta and advanced to the heart. When the manipulator is close enough, three flaps are opened, stabilizing the external part of the manipulator and allowing the internal structure with the new valve, named introducer, to advance. The introducer reaches the annulus, and the valve is rotated around the main axis of the manipulator to match the nadirs of the aortic cusps, as shown in Figure 4.

The surgery is carried out under the guidance of three cameras, positioned on the manipulator 120° from each other along a circumference of 21 mm in diameter. Microcameras (FisCAM, FISBA, Switzerland), 1.95 mm in diameter including illumination, were selected to fit into the reduced dimensions of the system. Illumination is given by LEDs from a separate control box, and it is directed through glass fibers. The specifics of the cameras are shown in Table 1.

#### 2.2.1. Image Stitching for Navigation to the Aortic Annulus

In this application case, the operatory site plane is the aortic annulus, which is the target of the surgical task. Since the exact position and orientation of the annulus plane cannot be estimated a priori, the homography is calculated considering a plane oriented parallel to the virtual camera.

**Figure 5:**
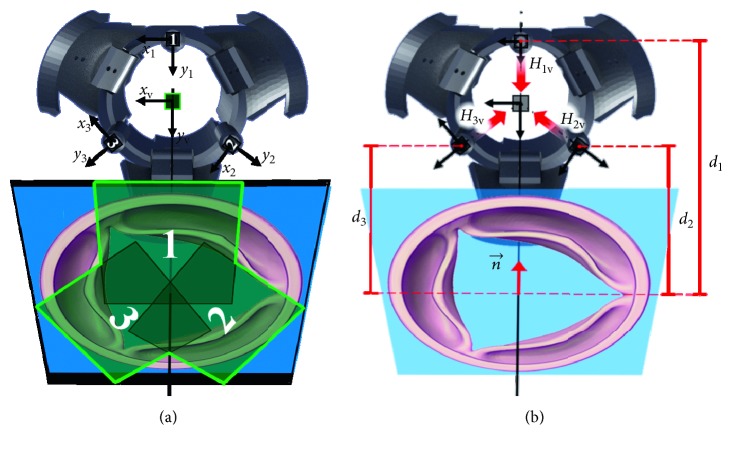
Basic components of homography and image stitching. (a) Manipulator with the flaps open: the reference systems of cameras 1, 2, and 3 and of the virtual camera (in green) are shown. The homographic plane is highlighted in blue, and the contributions of the three views, merged and captured by the virtual camera, are distinguished. (b) Manipulator with the flaps open: *H*_1_, *H*_2_, and *H*_3_ are the homographic transformations from the view of each camera to the virtual camera. Parameters *d*_1_, *d*_2_, and *d*_3_ represent the distance of each camera from the homographic plane. The vector normal to the plane, *n*, is unique; however, it assumes different values in the reference systems of the three cameras.

Figure 5(b) shows the plane normal vector, *n*, defined by this constraint, and the distance from the cameras reference system, *d*_*i*_. The plane distance *d*_1_ is set to 40 mm, corresponding to the average distance for valve releasing.  Figure 5(a) highlights the composition of the stitched images from the views from cameras 1, 2, and 3.

**Figure 6:**
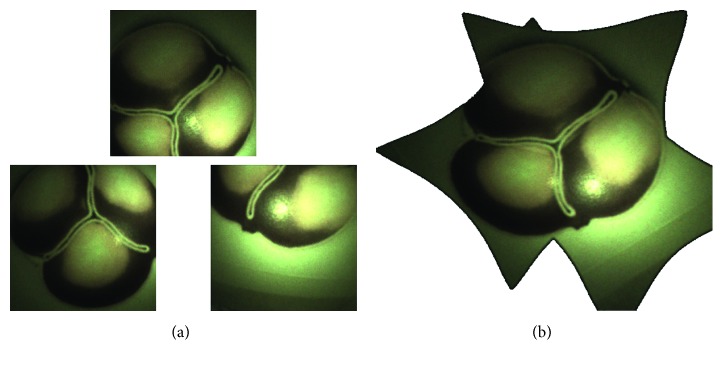
Single images and corresponding stitched image. The cameras capture a representation of a closed aortic valve at 40 mm. (a) Single views. (b) Stitching view: images are homographed and merged to reproduce the view from the virtual camera.

**Figure 7:**
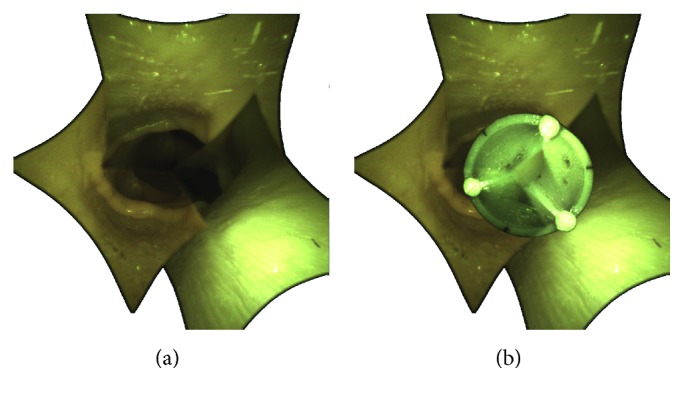
Basic concept of the AR support planned to ease the correct valve deployment. The release of the valve is simulated by showing in AR the final positioning the open valve, deployed from the current manipulator position [15].

Figure 6 shows on the left the single views of the three cameras. On the right, the result of the image stitching is shown; in this way, we obtain a single view that includes all the spatial information from the three cameras. The resulting stitched image can be enriched with virtual information content to provide the surgeon with an aid for the correct valve deployment.  Figure 7 illustrates the basic concept of an augmented reality (AR) aid which can be implemented to simulate the final positioning of the valve by knowing the position/orientation of the manipulator at the deployment time (this functionality requires that the release of the valve from the manipulator is repeatable and predictable).

### 2.3. Test

Quantitative test and qualitative test were performed to respectively evaluate the precision of the stitching procedure and the usability of the stitched endoscopic image for the navigation to the nadir point.

#### 2.3.1. Quantitative Test

**Figure 8:**
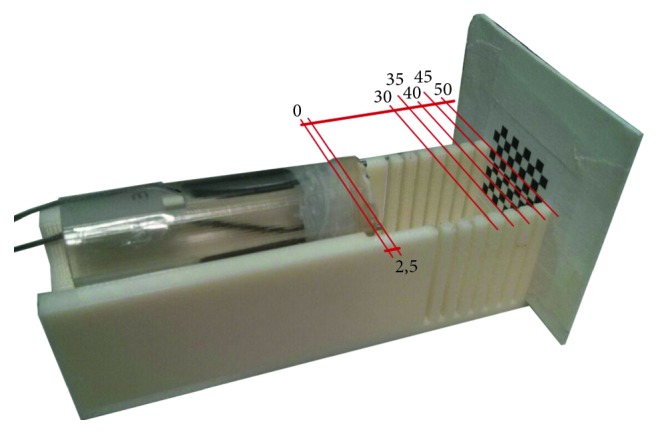
Support structure for quantitative error assessment. The tines are positioned so that the chessboard is parallel to the image plane of the virtual camera. Unit of measurement is in millimeters.

Quantitative measures aim at assessing the error in stitching the warped camera images. The error in terms of pixels was measured considering the misalignment of homologous features on pairs of warped images. Such error was evaluated respectively on a plane placed at 40 mm from the reference camera, i.e., the homography plane, and at incremental distances of 2.5 mm up to a depth of 20 mm. An 8 × 9 chessboard with a square side of 3 mm was used for the evaluation with the corners acting as reference features. In order to evaluate the mismatch introduced by the increasing distance from the homography plane, the chessboard was placed parallel to the image plane of the virtual camera by means of the support structure shown in Figure 8.

#### 2.3.2. Qualitative Usability Tests

Experimental tests were conducted to evaluate the usability of the stitched view during navigation in the aorta to the nadirs and while releasing the valve. Three cardiac surgeons already experienced in the AVR procedure via mini-thoracotomy and mini-sternotomy tested the view modalities.

**Figure 9:**
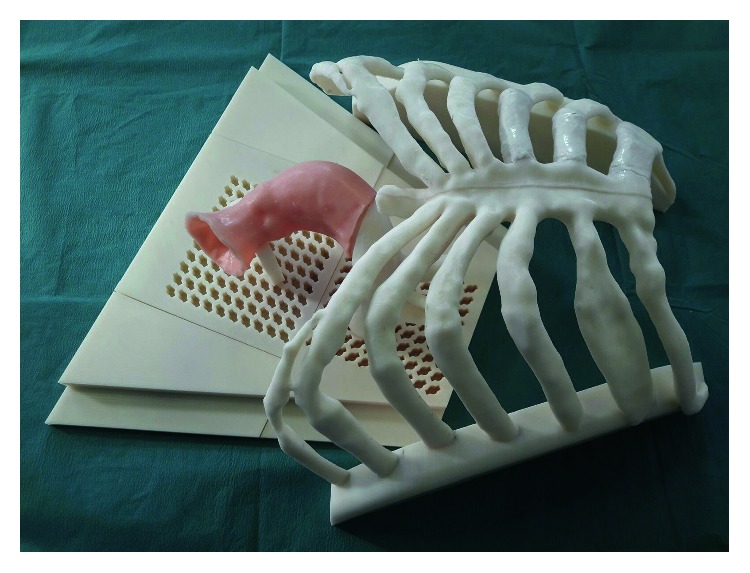
Patient-specific simulator used for qualitative usability tests. The tests were divided into two parts, I and II.

Test was performed by using the simulation setup developed in [11], which includes a patient-specific replica of the rib cage, aortic arch, ascending aorta, and the aortic valve, as shown in Figure 9. The aortic arch is made of ABS, and it is provided with a pin to anchor it to a base, while the ascending aorta and the aortic valve are made of soft silicone for a realistic interaction with surgical instruments with casting technique, as described in [29–31].

In Test I, the stitched view mode is compared with the three views' mode in relation to three key points:

- Ability to use display mode to orient within the aorta
- Ability to use display mode to navigate within the aorta
- Safety in releasing the valve using the display mode under examination

**Figure 10:**
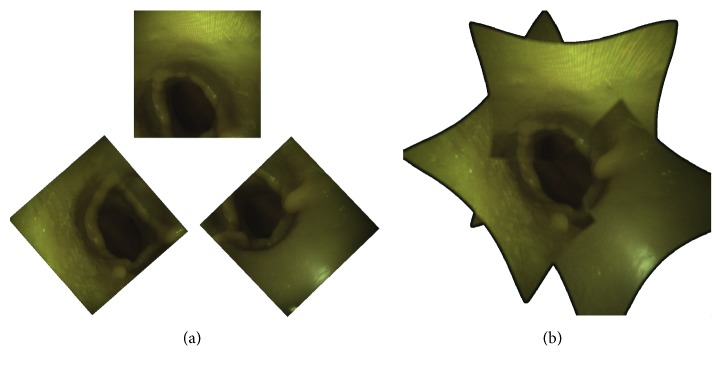
Comparison of view modes with and without stitching. (a) Single images view mode: the images from cameras 2 and 3 are rotated to align the horizon with that of the reference camera 1. (b) Stitching mode with semitransparent edges.

Upon request, each mode offers an augmented reality view of the valve positioning once released. To isolate the contribution of merging individual views into a single image, images of the three views' mode are prerotated. The rotation angle is such that the horizons of cameras 2 and 3 coincide with the horizon of reference camera 1. This result is achieved by decomposing the *R*_*i*_ matrices into Euler angles and rotating the images from cameras 2 and 3 according to the *Z*-axis angle.  Figure 10 shows the three views' mode with parallel horizon and the stitched view.

**Figure 11:**
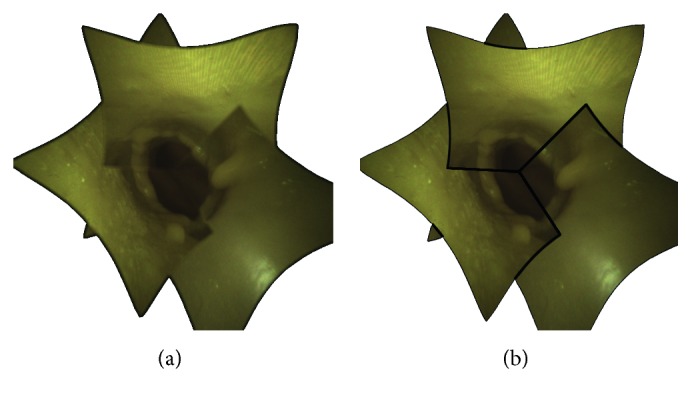
Comparison of smooth and clear transition in the stitched image. (a) Smooth transition through semitransparent edges. (b) Clear transition through black lines.

In Test II, the best way to manage transitions between images in the stitched view is investigated.  Figure 11 compares the two transition modes. One mode clearly demarcates the transition from one image to another through black lines. The other consists in a gradual transition between images, so that it is not possible to identify any borderline between them. The two transition modes were evaluated on the basis of the eye strain and the disturbance to navigation.

Surgeons were asked to navigate through the silicone aorta replica, reproducing the remains of the calcified aorta after removal. After completing the tests, surgeons filled out the questionnaire in Tables 2 and 3, organized in accordance with the 4-point Likert scale.

## 3. Results and Discussion

### 3.1. Quantitative Results

For each pair of warped images from cameras 1, 2, and 3, we computed the misalignment error in terms of mean and standard deviation between homologous corners of the chessboard pattern.  Table 4 shows the statistical parameters as a percentage of the warped image side.

**Figure 12:**
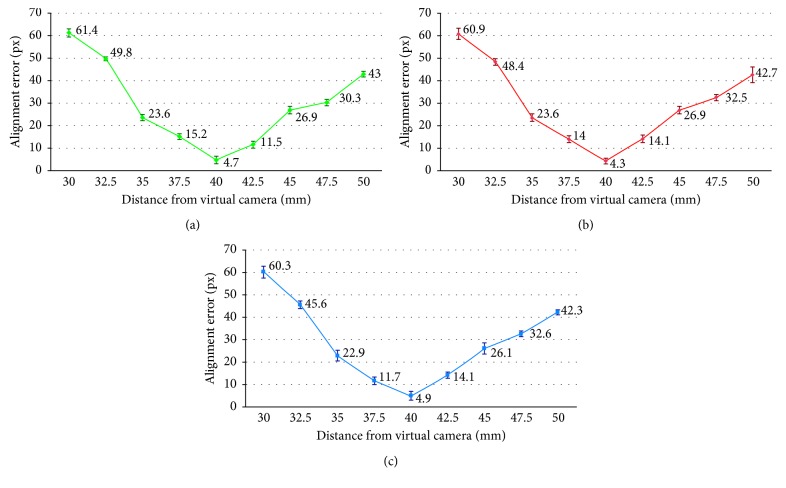
Error trend as the distance from the virtual camera varies. The error bars are expressed as twice the standard deviation. The number of samples varies in relation to the number of corners that can be identified in the images, and it is included in the range 32–56. Alignment error cameras: (a) 1-2, (b) 2-3, and (c) 1–3.

**Figure 13:**
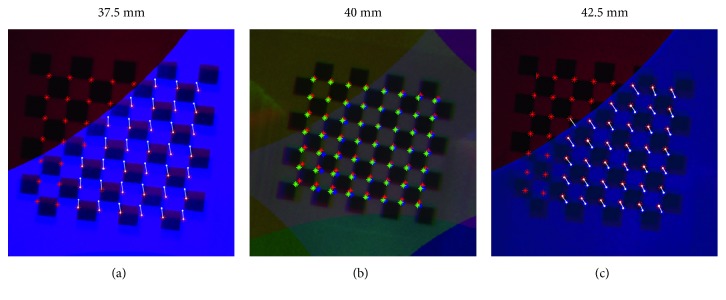
Error in alignment by moving away from the homographic plane placed at 40 mm. (a, c) The images of the reference camera are compared with the images of camera 2 at 37.5 mm and 42.5 mm, respectively. The distances between homologous pixels of the two cameras are highlighted in white. (b) The images acquired by the three cameras are compared, showing in red the chessboard corners from the reference camera, in blue the corners from camera 2, and in green the corners from camera 3.

Plots in Figure 12 show the error trend in px. By way of an example, Figure 13 illustrates the misalignment between the camera images at distances of 37.5 mm, 40 mm, and 42.5 mm. The quantitative results show an increase in error when moving away from the homography plane. However, the constraints imposed by the specific surgical task and by the limited area surrounding the aorta significantly restrict the working area. Specifically, although the manipulator can adapt to tortuous and narrow paths through its link segments, the field of view of the cameras will always be obstructed by any curves. As a result, the length of the ascending aorta, which is about 5 cm [32], limits the maximum distance captured by the camera. Also, being the optimal position of the manipulator usually 3-4 cm away from the calcified valve [26], when positioning the valve, the translation along the aorta axis is limited to 1-2 cm. In this area, the maximum misalignment of 6.09 ± 0.23% is negligible compared to the size of the stitched image. This error is further decreased as the system approaches the optimal alignment, and it is reduced to 0.47 ± 0.16% at the homography plane. Therefore, in the most delicate phase of the operation, i.e., during the valve releasing, the error is kept to a minimum.

### 3.2. Qualitative Usability Results

Test results are expressed as median of the assessments.  Table 5 shows that the three views' mode and the stitching view mode do not differ in terms of ability to orient and to navigate within the aorta. However, the stitching view provides surgeons with greater safety when releasing the valve. This may be due to the feasibility of orientation and navigation within the aorta even through the guidance of a single camera and eventually shifting the gaze to different views when necessary. But, when releasing the valve, an iterative alignment on the different views is more complex than simultaneously checking the match with all nadirs from a single image.  Table 6 describes the results of the comparison between black borders and blurred borders transition mode, highlighting a preference for blurred border mode.

Tests were completed on a laptop with CPU 2.0 GHz processor, 8 GB RAM, and Windows 8.1 as an operating system. Experiments show that the proposed approach is effective in terms of computational complexity: time taken to stitch 3 images was of 12.6 ms averaged over an 8-minute video. Computational time is one of the most important parameters for measuring the stitching performance. The proposed method has low computational cost, as it does not require algorithms for the identification of common features in the images: experimental tests conducted with similar hardware shows that methods based on features detectors (Harris corner detector, SIFT, SURF, FAST, goodFeaturesToTrack, MSER, and ORB techniques) require from 60 ms up to 1.2 s for images with a lower resolution (320 × 225) only for detecting features [33], and the total computational time is further increased by computing and applying the image transformation.

## 4. Conclusions

The article proposes a method for merging the images acquired by three cameras into a single image that encompasses their single contributions. The cameras are placed off-center with respect to the axis of the manipulator, and the stitched image restores a central view to the user. The proposed method has low computational cost, as it does not require algorithms for the identification of common features in the images, but it is based on the knowledge of the reciprocal poses between the cameras and on the position and orientation of a reference plane in space. Taking advantage of the constraints imposed by the specific surgical procedure and by the aorta conformation, plane-induced homographies are used to merge the camera views. Quantitative tests showed that, although the misalignment grows moving from the homography plane, it remains negligible compared to the image size. Experimental tests with surgeons confirmed these results; they showed that the stitched view, allowing the visualization of the three nadir points in a single image, would allow surgeons to release the valve more safely, while not compromising orientation and navigation in the vessel.

## Figures and Tables

**Table 1:** Technical specifications of the FisCAM cameras.

FisCAM, FISBA, Switzerland	
Resolution (px)	400 × 400 at 30 fps
Working distance (mm)	5–50
Diagonal field of view (degrees)	120°

**Table 2:** 4-point Likert scale questionnaire for comparison between single views and stitched view mode.

The view allows to easily orient inside the aorta.
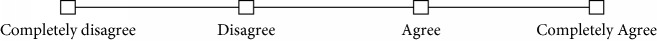
It is possible to navigate the anatomy, visualizing the three nadirs.
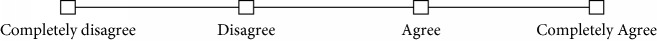
This display mode would allow the valve to be released safely.
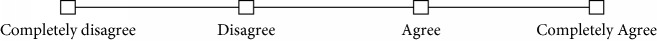

**Table 3:** 4-point Likert scale questionnaire for comparison between black line transitions and blurred transitions.

Black lines\blurring marking the transition between images, do not strain your eyes during navigation.
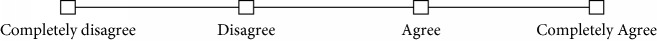
Black lines\blurring marking the transition between images, do not disturb navigation.
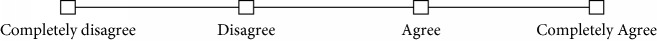

**Table 4:** Statistical parameters of the mismatch error as the distance from the reference camera varies.

Distances from virtual camera (mm)	Cameras 1-2		Cameras 1–3		Cameras 2-3		Cameras' mean	
	Mean value (%)	Standard deviation (%)	Mean value (%)	Standard deviation (%)	Mean value (%)	Standard deviation (%)	Mean value (%)	Standard deviation (%)
30	6.14	0.18	6.03	0.26	6.09	0.25	6.09	0.23
32.5	4.98	0.09	4.56	0.18	4.84	0.13	4.79	0.13
35	2.36	0.15	2.29	0.23	2.36	0.17	2.34	0.18
37.5	1.52	0.13	1.17	0.17	1.4	0.14	1.36	0.15
40	0.47	0.16	0.49	0.19	0.43	0.13	0.46	0.15
42.5	1.15	0.15	1.41	0.14	1.41	0.17	1.32	0.15
45	2.69	0.16	2.61	0.25	2.69	0.16	2.66	0.19
47.5	3.03	0.14	3.26	0.12	3.25	0.13	3.18	0.13
50	4.30	0.12	4.23	0.11	4.27	0.35	4.27	0.19

For each distance from the reference camera, the average value as a percentage of the homographed image side and the error standard deviation are reported. Values are given for each pair of cameras, 1-2, 1–3, and 2-3, and finally as an average of the three camera pairs.

**Table 5:** Results of the questionnaire comparing the three views' mode with the stitching view mode.

	Three views	Stitching
The view allows to easily orient inside the aorta	3 (agree)	3 (agree)
It is possible to navigate the anatomy, visualizing the three nadirs	3 (agree)	3 (agree)
This display mode would allow the valve to be released safely	3 (agree)	4 (completely agree)

**Table 6:** Results of the questionnaire comparing the black-edged and blurred-edged transition mode.

	Black lines	Blurring
Transitions between images do not strain your eyes during navigation	2 (disagree)	3 (agree)
Transitions between images do not disturb navigation	2 (disagree)	3 (agree)

## Data Availability

The data used to support the findings of this study are available from the corresponding author upon request.
